# Plasma and Nail Zinc Concentrations, But Not Hair Zinc, Respond Positively to Two Different Forms of Preventive Zinc Supplementation in Young Laotian Children: a Randomized Controlled Trial

**DOI:** 10.1007/s12011-020-02163-2

**Published:** 2020-04-30

**Authors:** K. Ryan Wessells, Kenneth H. Brown, Charles D. Arnold, Maxwell A. Barffour, Guy-Marino Hinnouho, David W. Killilea, Sengchanh Kounnavong, Sonja Y. Hess

**Affiliations:** 1 Institute for Global Nutrition, Department of Nutrition, University of California, Davis, One Shields Ave, Davis, CA 95616 USA; 2 McQueary College of Health and Human Services, Public Health Program, Missouri State University, 606E Cherry St, Springfield, MO 65897 USA; 3 Children’s Hospital of Oakland Research Institute, 5700 Martin Luther King Jr Way, Oakland, CA 94609 USA; 4 Lao Tropical and Public Health Institute, Ban Kaognot, Sisattanak District, Vientiane, Lao People’s Democratic Republic

**Keywords:** Zinc, Biomarker, Supplement, Nail, Hair, Plasma

## Abstract

Plasma zinc concentrations (PZC) have been shown to significantly increase during zinc supplementation. This study investigated the effects of daily preventive zinc supplementation on hair and nail zinc concentrations compared with a control group. In a randomized controlled trial, 6- to 23-month-old children (*n* = 3407) in Lao PDR were randomly assigned to one of four groups and followed for ~ 36 weeks: daily preventive zinc dispersible tablet (7 mg/d; PZ), daily micronutrient powder (10 mg zinc/d; MNP), therapeutic zinc supplements for diarrhea treatment (20 mg/d for 10 days; TZ), or daily placebo powder (Control). Plasma, hair, and nail zinc concentrations were assessed in a sub-sample of participants (*n* = 457) at baseline and endline. At baseline, 75% of children had low PZC (< 65 μg/dL). At endline, geometric mean (95% CI) PZC were greater in the PZ and MNP groups compared with the TZ and control groups (*P* < 0.01), but hair zinc concentrations did not differ among groups (*P* = 0.99). Nail zinc concentrations were marginally higher in the PZ (115.8 (111.6, 119.9) μg/g) and the MNP (117.8 (113.3, 122.3) μg/g) groups than in the TZ group (110.4 (106.0, 114.8) μg/g; *P* = 0.055) at endline. This study does not support the use of hair zinc as a biomarker of zinc exposure in young children. However, it provides some evidence that zinc concentrations in nails may respond to supplemental zinc interventions and supports the need for collecting additional data on this emerging biomarker.

**Electronic supplementary material:**

The online version of this article (10.1007/s12011-020-02163-2) contains supplementary material, which is available to authorized users.

## Introduction

Zinc is an essential micronutrient for human health, and infants and young children in low- and middle-income countries are at high risk of zinc deficiency [1, 2]. Preventive zinc supplementation has been shown to decrease morbidity from diarrhea and acute lower respiratory infections, reduce all-cause mortality, and increase linear growth and weight gain among infants and young children in vulnerable populations [3]. In addition, therapeutic zinc supplementation administered during episodes of diarrhea has been shown to reduce the duration of diarrheal illness [4].

Recent recommendations from the Biomarkers of Nutrition for Development (BOND) zinc expert panel, the WHO, UNICEF, International Atomic Energy Agency (IAEA), the International Zinc Nutrition Consultative Group (IZiNCG), and the EURopean micronutrient RECommendations Aligned (EURRECA) Network of Excellence [5–7] have concluded that plasma zinc concentration is the only valid biochemical indicator currently available to assess the risk of zinc deficiency in populations and measure population-level exposure to zinc supplementation. Significant increases in individual and population mean plasma zinc concentrations have been consistently observed following zinc supplementation, regardless of initial concentrations of plasma zinc [6]. In addition, reference data are available to define zinc deficiency based on plasma zinc concentrations [8]. However, factors independent of zinc status (e.g., fasting, diurnal and circadian rhythms, age, sex, and infection and inflammation) can influence plasma zinc concentrations and thus can confound the interpretation of zinc status [6]. Moreover, the measurement of plasma zinc concentration requires invasive blood collection, biohazardous materials, complex preparation, and consideration of downstream processes like transportation and storage of samples, including cold chain control [9].

Two alternative biochemical measures of zinc exposure and status, namely, hair and nail zinc concentrations, were recently classified by the BOND zinc expert panel as potential and emerging biomarkers, respectively [5, 10]. The concentrations of zinc in hair and nails are thought to reflect the amount of zinc in the blood supply at the time of their synthesis. Hair zinc concentrations have been shown to increase following zinc supplementation among adults [7]; however, its response to supplemental zinc has been inconsistent among infants and young children [5]. Evidence for the use of nail zinc concentrations as an indicator of zinc response to supplementation is even more limited, in part due to the previous lack of sensitive measurement techniques [5].

The assessment of zinc concentrations in hair and nails has several theoretical advantages compared with plasma, including non-invasive collection, greater stability of specimens, and lower sensitivity to physiologic confounders (i.e., diurnal variation, circadian rhythm, inflammation). However, additional research is needed on the response of hair and nail zinc concentrations to supplemental zinc intake among infants and young children, and reference data must be collected to define normal ranges before these indicators can be recommended for the assessment of zinc status.

To help fill the first of the aforementioned data gaps, the primary objective of the present study was to investigate the effects of daily preventive zinc supplementation, provided either as dispersible zinc tablets or low-iron high-zinc containing multiple micronutrient powders (MNP), on changes in hair and nail zinc concentrations compared with a control group. A secondary objective was to evaluate the relationships between plasma zinc concentrations and hair and nail zinc concentrations as alternative biomarkers of zinc status.

## Materials and Methods

### Study Design and Participants

The current analyses are based on sub-sample of participants enrolled in a community-based randomized, double-blind, placebo-controlled intervention trial. The main trial was designed to compare two forms of daily preventive zinc supplementation (7-mg/d zinc as zinc sulfate, provided as a dispersible tablet or 10-mg/d zinc as zinc gluconate, provided in a multiple micronutrient powder) versus therapeutic zinc supplementation (20-mg/d zinc as zinc sulfate, provided for 10 days in relation to an episode of diarrhea) or placebo on young children’s physical growth and risk of infection over the 36-week intervention period. Detailed descriptions of the overall study design, as well as the effect of the interventions on the primary outcomes (i.e., physical growth, diarrhea incidence, hemoglobin and micronutrient status, and innate and adaptive immune response) have been reported previously [11–14]. The current analyses address secondary outcomes of the trial.

The study was conducted in five districts within Khammouane Province of the Lao People’s Democratic Republic (PDR) from September 2015–April 2017. In total, 3407 children were enrolled. Children were eligible to enroll in the study if they met the following inclusion criteria: aged 6 to 23 months, intended residence in the study catchment area for the duration of the study, acceptance of weekly home visits, and signed informed consent from a parent or guardian. Exclusion criteria were severe anemia (hemoglobin < 70 g/L), severe acute malnutrition (weight-for-length z-score (WLZ) < − 3 SD and/or bipedal edema), serious health condition requiring medical attention, current consumption of zinc supplements, or current participation in another research study.

The trial protocol was approved by the Institutional Review Board of the University of California, Davis (IRB; 626187) and the National Ethics Committee for Health Research, Ministry of Health, Lao PDR (NEHCR; 040/2014, 069/2015, 039/2016). The study was registered as a clinical trial (www.ClinicalTrials.gov; NCT02428647). Consent materials were presented both orally and written in Lao language, in the presence of an impartial witness. Informed consent, documented with either a written signature or a fingerprint, was obtained from a parent or guardian of each child before his or her enrollment in the study.

### Randomization and Intervention Products

Detailed information regarding randomization and blinding procedures, supplement composition, instructions provided to caregivers, and distribution and monitoring of adherence has been published previously [11]. In brief, participants were individually randomized to one of four intervention groups, using a computer-generated, block-randomization list prepared by a statistician from the University of California, Davis. The intervention groups were as follows: (1) daily preventive zinc supplement tablets, containing 7-mg of zinc and placebo tablets for diarrhea (PZ group); (2) daily preventive MNP supplements, containing 10-mg zinc, 6-mg iron, and 13 other micronutrients, and placebo tablets for diarrhea (MNP group); (3) daily placebo preventive supplements and therapeutic zinc tablets, containing 20-mg zinc for 10 days for diarrhea treatment (TZ group); and 4) daily placebo preventive powder and placebo tablets for diarrhea (Control group). Diarrhea kits were stored in the children’s homes, to be immediately available for treatment of diarrhea; each kit contained 10 tablets of the group-specific form of the dispersible therapeutic tablet, as well as low-osmolarity oral rehydration salts and written and pictorial instructions. The preventive zinc tablets were produced by Nutriset SAS (Malaunay, France). The MNP and placebo powder were produced by DSM Fortitech Asia Pacific Sdn Bhd (Banting, Malaysia); nutrient content is presented in Supplemental Table 1. For the duration of the intervention (~ 36 weeks), the households were visited weekly by a field worker who delivered the child’s assigned preventive supplements, replaced any used diarrhea kits, assessed adherence to supplementation, and conducted a systematic, symptom-based morbidity recall history.

### Structured Interviews and Anthropometry

Information on infant and young children feeding practices was collected [15] and demographic and socioeconomic characteristics of the participants’ households (e.g., maternal age and education, and household food insecurity (HFIAS) [16], assets, and hygiene and sanitation indicators) were assessed via structured interview at baseline.

Children’s weight and recumbent length were measured in duplicate at baseline and endline (32–40 weeks), following the procedures recommended by the Food and Nutrition Technical Assistance project [17]. Z-scores for weight-for-age (WAZ), length-for-age (LAZ), and weight-for-length (WLZ) were calculated in relation to the WHO growth standards [18]. Underweight, stunting and wasting were defined as LAZ, WAZ, and WLZ < −2 SD, respectively. Maternal height and weight were measured in duplicate at one time point during the study, usually at baseline [11].

### Biological Sample Collection

Biological samples were collected and processed according to procedures recommended by the IZiNCG [9, 10]. Venous blood was collected at baseline and endline (~ 32–36 weeks) in 7.5-ml evacuated, trace element-free, polyethylene tubes containing lithium heparin (Sarstedt AG & Co, Numbrecht, Germany) and stored at 4–8 °C until separation of plasma within 8 h. Blood samples were centrifuged (PowerSpin Centrifuge Model LX C856; United Products & Instruments, Inc., Dayton, NJ) at 1097×g for 10 min, and plasma aliquots were stored at − 20 °C until shipment on dry ice to the University of California, Davis. Also at baseline and endline, hair (10–20 mg) was cut as close to the scalp as possible, from the center of the nape of the neck (~ 2 cm above the hair line). In the case of insufficient hair at the nape of the neck, samples were obtained from a nearby location, working upward toward the crown until a sufficient sample could be achieved. In the case of longer hair (> 2 cm), the cut sample was taped to an index card using cellophane tape, with the side closest to the scalp identified. At endline only, finger and toenails were cut using infant safety nail clippers. All samples were placed in small paper envelopes and stored at ambient temperature within Ziploc bags containing desiccants. Nail samples from each child’s fingers and toes were pooled together because of the small mass of available material.

### Biological Samples Analysis

Zinc content was measured in plasma, hair, and nail samples. For plasma, samples were thawed, vortexed for 5–10 s, and then directly aliquoted into 100-μl samples for analysis. For hair, multiple hair segments were trimmed to 1- to 2-cm pieces without the root and were placed into microcentrifuge tubes to reach a target mass of 3–10 mg. For nails, multiple nail fragments were manually scraped with a ceramic blade to remove debris and placed into microcentrifuge tubes to reach a target mass of 3–10 mg. Hair and nail samples were then cleaned to remove external zinc contamination using a modified version of the cleaning protocol recommended by IZiNCG [10]. Cleaning started with three cycles of sonication in 2 ml of 1% Triton X-100 (Sigma-Aldrich, St. Louis, MO) for 10 min, then sonication in 2 ml of OmniTrace water (VWR International, West Chester, PA) for 1 min, then sonication in 2 ml of 100% acetone (Sigma-Aldrich) for 10 min, ending with drying at 40 °C for 12–18 h. After drying was complete, hair and nail samples were carefully weighed to measure dry weight; hair and nail sample weights were determined to be 3.4 + 1.1 mg and 3.0 + 1.2 mg, respectively. Then the plasma, hair, and nail samples were digested in OmniTrace 70% HNO_3_ (VWR International) for 12–18 h. Hair and nail samples were completely dissolved with this protocol. All samples were then diluted to a final concentration of 5% HNO_3_ and centrifuged at 3000×*g* for 10 min prior to analysis using inductively coupled plasma optimal emission spectrophotometry (5100 ICP-OES SVDV, Agilent, Santa Clara, California) as described previously [12, 19]. Plasma zinc content was normalized to sample volume, while hair and nail zinc content was normalized to samples’ dry weight. Seronorm Trace Elements Serum L-1 and L-2 (Accurate Chemical and Scientific Corp, Westbury, New York) and Hair Certified Reference Standard ERM-DB001 (Sigma-Aldrich) reference materials were prepared according to manufacturer specification and analyzed in an identical manner to that of the clinical samples. Plasma C-reactive protein (CRP) and α-1-acid glycoprotein (AGP) concentrations were measured using combined sandwich enzyme-linked immunosorbent assay (ELISA) technique at the VitMin Lab (Willstaett, Germany), as described previously [20].

### Sample Size Estimation

The main trial enrolled 3407 children; of these, 760 children were included in the biochemical outcomes sub-sample (*n* = 190 per group). Due to logistical challenges caused by a large study catchment area, the biological sub-sample included only children who were enrolled in the study from the two health districts closest to the study office, until the sample size was met [11]. All children from the biochemical sub-sample were eligible to be included in the present analyses if they were randomized to the PZ, MNP, or control intervention group, with preference given to children who met the following criteria: (1) plasma samples available from baseline and endline, with data available for plasma zinc concentration, CRP and AGP; (2) baseline and endline hair samples available, with data available for hair cortisol; and (3) endline nail sample available. The group receiving therapeutic zinc supplementation for diarrhea was not intended to be included in these secondary analyses because there was no effect of intermittent short-course zinc supplementation on plasma zinc concentrations [12], so the effects on hair and nail zinc concentrations were assumed to be limited. However, due to an administrative error that occurred while study personnel were still blinded to the treatment group, nail zinc samples from the TZ intervention group were analyzed in lieu of those from the control group. Therefore these results should be considered exploratory.

The available sample size of ~ 123 participants per group with analyzed baseline and endline hair samples and ~ 83 with endline nail samples allows detection of treatment-related differences in hair and nail zinc concentrations (primary outcomes) having an effect size of 0.40–0.50 SD with 80% power and a 5% level of significance. The available sample size permits detection of relationships with a strength of correlation (r) of at least 0.18 between plasma zinc concentration and the alternative biomarkers of zinc exposure and status (hair and nail zinc concentrations).

### Statistical Analyses

A detailed statistical analysis plan was developed prior to analysis and is available online [21]. All analyses were completed using a complete-case, intention-to-treat approach [22]. Descriptive statistics were calculated for all variables. Model assumptions were assessed (e.g., Shapiro-Wilk tests for normality and assessments of linearity) and variables were appropriately transformed prior to further analysis. Plasma zinc concentrations were adjusted for inflammation (elevated CRP and/or AGP), using procedures adapted from the Biomarkers Reflecting Inflammation and Nutritional Determinants of Anemia (BRINDA) project and described in detail elsewhere [12, 23]; results are presented for inflammation-adjusted concentrations.

Differences in mean hair, nail, and plasma zinc concentrations by treatment group after the intervention were assessed by ANCOVA. Minimally adjusted models controlled for the baseline value of the dependent variable (if available), age at enrollment and health district. When there was a significant treatment effect by likelihood ratio test (*P* < 0.05), pairwise comparisons were performed to assess differences between groups. The same methods were repeated for fully adjusted ANCOVA models, where prespecified covariates determined to be associated with an outcome (*P* < 0.10) in a bivariate analysis were included in the final adjusted model. Prespecified potential effect modifiers were assessed by including an interaction term in the ANCOVA model; significant interactions (*P* < 0.10) are reported and examined with stratified analyses in order to understand the nature of the effect modification.

Associations between plasma zinc concentrations and hair zinc concentrations at baseline were examined with linear regression. In addition, associations between plasma zinc concentrations and (1) hair and (2) nail zinc concentrations were assessed at endline, controlling for treatment group. All analyses were adjusted for age at enrollment and health district. The methods described above for fully adjusted models and the assessment of potential effect modifiers were repeated in these linear regression models.

Zinc deficiency was defined as a plasma zinc concentration < 65 μg/dL [24]. Low hair zinc level was defined as a hair zinc concentration < 70 μg/g hair [5, 10]. Low nail zinc level has not been established. Data were analyzed in R version 3.5.0. Data are presented as means + SD or median (IQR), unless otherwise noted. The investigators remained blinded to treatment groups until all primary analyses were completed.

## Results

### Participant Characteristics

**Fig. 1:**
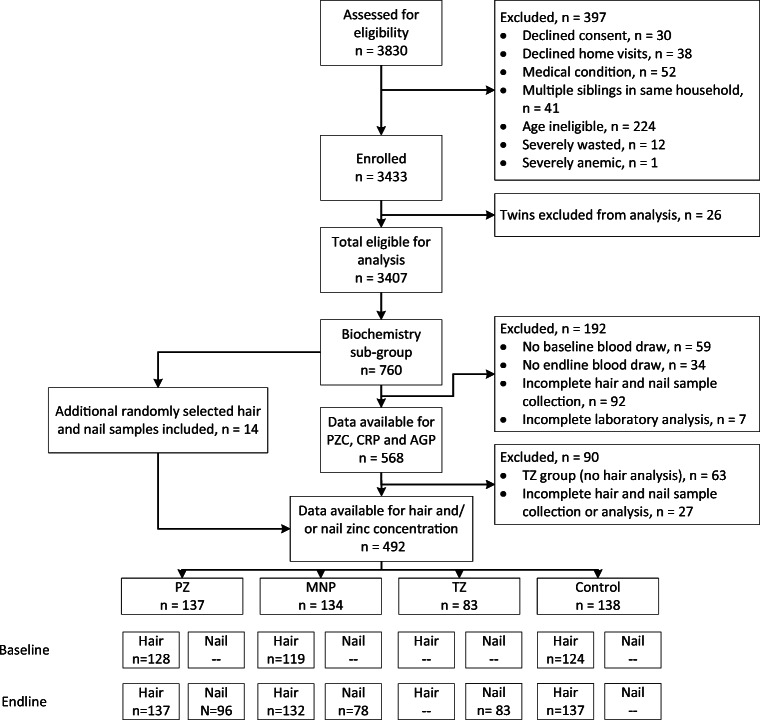
Flowchart of participant progression through the randomized controlled trial In total, 492 children were included in analyses. All children for whom baseline and endline hair Zn concentrations were available (*n* = 368), or for whom endline nail zinc concentrations were available (*n* = 256), were included in the analyses of the impact of the intervention (primary objective). Among these children, 11 did not have analyzed plasma zinc concentrations and were thus not included in analyses investigating correlations between biomarkers (secondary objective). However, an additional 34 children who had measurements of baseline or endline hair zinc concentration, in addition to plasma zinc concentrations, were included in at least one of the correlational analyses (secondary objective).

A total of 492 children who provided at least one hair or nail sample are included in the present analyses (Fig. 1). Characteristics of those children with baseline and endline hair samples or endline nail samples, and thus contributing to the primary outcome analyses, are presented in Table 1**.** The mean age at baseline was 15.9 ± 5.0 months. The prevalence of stunting and wasting were 37.9% and 6.1%, respectively. At baseline, the median plasma zinc concentration was 56.4 (49.3, 65.0) μg/dL and 75.2% of children had low plasma zinc concentrations (< 65 μg/dL). Median hair zinc concentration at baseline was 54.7 (39.1, 78.9) μg/g; 66.2% of children had low hair zinc concentration (< 70 μg/g). Hair zinc concentration at baseline was not associated with age (*P* = 0.735) but was associated with sex, with females having a lower median hair zinc concentration than males (45.1 μg/g vs. 67.5 μg/g; *P* < 0.001).

**Table 1:** Child, maternal, and household characteristics of the study participants at baseline by intervention group^a^

Variables	All	PZ	MNP	TZ	Control
*N*^b^	457	128	123	83	123
Age, months^c^	15.9 ± 5.0	15.6 ± 5.2	16.0 ± 5.0	16.8 ± 4.6	15.5 ± 4.9
District					
Nongbok	151 (33.0)	46 (35.7)	36 (29.3)	26 (31.3)	43 (35.0)
Xebangfai	307 (67.0)	83 (64.3)	87 (70.7)	57 (68.7)	80 (65.0)
Male	207 (45.2)	50 (38.8)	61 (49.6)	41 (49.4)	55 (44.7)
Anthropometry					
LAZ	− 1.70 ± 1.10	− 1.77 ± 1.17	− 1.78 ± 1.05	− 1.91 ± 1.03	− 1.41 ± 1.09
Stunted (LAZ < −2 SD)	173 (37.9)	51 (39.8)	49 (39.8)	37 (44.6)	36 (29.3)
WAZ	− 1.41 ± 1.00	− 1.51 ± 1.00	− 1.48 ± 1.03	− 1.58 ± 0.99	− 1.13 ± 0.95
Underweight (WAZ < − 2 SD)	126 (27.6)	40 (31.3)	38 (30.9)	28 (33.7)	20 (16.3)
WLZ	− 0.74 ± 0.86	− 0.82 ± 0.87	− 0.78 ± 0.87	− 0.84 ± 0.85	− 0.56 ± 0.82
Wasted (WLZ < − 2 SD)	28 (6.1)	10 (7.8)	10 (8.1)	6 (7.2)	2 (1.6)
Biochemical Indicators					
Plasma zinc concentration, μg/dL^d^	56.4 (49.3, 65.0)	55.2 (47.9, 65.6)	57.4 (50.1, 65.9)	57.2 (51.0, 65.0)	56.4 (49.6, 63.1)
Plasma zinc concentration < 65 μg/dL	336 (75.2)	93 (72.7)	89 (73.0)	62 (75.6)	92 (80.0)
Hair zinc concentration, μg/g	54.7 (39.1, 78.9)	52.6 (35.8, 77.7)	60.3 (42.8, 78.9)	–	52.0 (38.5, 83.3)
Hair zinc concentration < 70 μg/g	245 (66.2)	88 (68.8)	77 (64.7)	–	80 (65.0)
C-reactive protein (CRP), mg/L	0.47 (0.21, 1.65)	0.55 (0.19, 1.70)	0.39 (0.23, 1.38)	0.42 (0.19, 1.61)	0.51 (0.22, 2.46)
CRP > 5 mg/L	55 (12.1)	16 (12.6)	10 (8.1)	11 (13.4)	18 (14.6)
α-1-acid glycoprotein (AGP), g/L	0.60 (0.44, 0.87)	0.62 (0.43, 0.86)	0.60 (0.45, 0.87)	0.59 (0.46, 0.85)	0.60 (0.44, 0.97)
AGP > 1 g/L	96 (21.1)	28 (22.1)	25 (20.3)	15 (18.3)	28 (22.8)
IYCF practices^e^					
Breastfed, in the previous month	258 (62.8)	69 (61.6)	66 (61.7)	48 (63.2)	75 (64.7)
Adequate dietary diversity	155 (37.6)	41 (36.6)	38 (35.2)	25 (32.9)	51 (44.0)
Minimum meal frequency	257 (62.5)	68 (60.7)	66 (61.7)	46 (60.5)	77 (66.4)
Maternal characteristics					
Maternal age, years	26.8 ± 6.1	26.3 ± 5.9	27.7 ± 6.4	27.2 ± 6.1	26.3 ± 5.8
Maternal BMI, kg/m^2^	22.0 ± 3.3	21.8 ± 3.1	22.2 ± 3.2	22.0 ± 3.7	22.2 ± 3.5
Maternal education, completed primary school	139 (31.0)	36 (28.8)	41 (33.3)	19 (23.5)	43 (36.1)
Household characteristics^d^					
HFIAS, moderately or severely food insecure	207 (45.9)	57 (45.2)	49 (40.8)	49 (59.0)	52 (42.6)
Latrine access	260 (63.0)	75 (67.0)	69 (63.9)	43 (65.6)	73 (62.4)
Use of improved drinking water source	304 (84.0)	81 (78.6)	85 (91.4)	51 (81.0)	87 (84.5)

^a^*AGP* α-1-acid glycoprotein, *BMI* body mass index, *CRP* C-reactive protein, *HFIAS* household food insecurity access scale [25], *LAZ* length-for-age z-score, *MNP* micronutrient powder, *PZ* preventive zinc, *TZ* therapeutic zinc, *WAZ* weight-for-age z-score, *WLZ* weight-for-length z-score

^b^Baseline characteristic data are presented for children included in at least one of the primary outcomes (i.e., data were available for baseline and endline hair zinc concentrations and/or endline nail zinc concentrations). Breastfeeding and minimum meal frequency, *n* = 411; adequate dietary diversity, *n* = 412; latrine access, *n* = 413; maternal BMI, improved drinking water source, *n* = 362

^c^Values presented as mean ± SD, *n* (%) or median (IQR)

^d^Plasma zinc concentrations are adjusted for elevated acute phase proteins (CRP and AGP) based on Barffour et al. [12], and estimates of the prevalence of deficiency are based on inflammation-adjusted concentrations

^e^Infant and young child feeding practices defined by the WHO [15]. HFIAS from the Food and Nutrition Technical Assistance III Project [16]. Socioeconomic status index based on available indicators of household socioeconomic status, education, income, and ownership of assets, land, and animals. Handwashing defined as report of consistent handwashing after defecation and/or before meal preparation compared with occasional or no handwashing after defecation and before meal preparation. Improved drinking water source defined by the WHO [26]

Over the course of the 9-month intervention, reported adherence to the daily preventive supplement (i.e., tablet, MNP, or placebo) was 90% and did not differ by intervention group (*P* = 0.375). The median number of 20 mg therapeutic zinc supplements consumed in the TZ group was 7 (range = 0–48), given during the ~ 237-day observation period, resulting in median zinc consumption of 140 mg (range = 0–960 mg).

### Effects of the Intervention

As reported previously, the mean endline plasma zinc concentrations were significantly higher among children in the PZ and MNP groups than in the control group [12]. This remained statistically significant in the sub-sample of children with hair zinc concentration data (*P* < 0.001) (Table 2), resulting in a significantly lower prevalence of zinc deficiency post-intervention in the PZ and MNP groups compared with the control group (53.9–61.9% vs. 75.6%, *P* = 0.001). However, in both the minimally adjusted (adjusted for just the baseline value of the outcome of interest, age, and health district) and fully adjusted models, the mean endline hair zinc concentrations did not differ among the three groups (Table 2).

**Table 2:** Effects of 32–40 weeks of supplementation with daily preventive zinc or daily multiple micronutrient powder on endline hair and plasma zinc concentrations among young Lao children^a^

Outcome	PZ		MNP		Control		Minimally adjusted *p* value^b^	Adjusted *p* value
	n	Value^c^	n	Value	n	Value		
Plasma zinc concentration, μg/dL^d^	124	65.8 (63.2, 68.5)	117	61.7 (59.1, 64.3)	115	55.2 (52.9, 57.5)	< 0.001	
Hair zinc concentration, μg/g	127	55.5 (51.1, 60.4)	118	56.0 (51.3, 61.1)	123	55.4 (50.9, 60.4)	0.987	0.999

^a^MNP, multiple micronutrient powder; PZ, preventive zinc

^b^Minimally adjusted and adjusted models control for age and district of enrollment. Adjusted models include child sex and maternal education

^c^Values represent mean (95% CI)

^d^Plasma and hair zinc concentration data control for baseline values of the respective outcome and plasma zinc concentrations are adjusted for elevated acute phase proteins (CRP and AGP) based on Barffour et al. [12]. Plasma zinc concentration data are only presented for children for whom hair zinc concentration data are also available

As noted above, the effects of the intervention on nail zinc concentrations were compared for the PZ and MNP groups versus the TZ group. In the sub-sample of children with nail samples, plasma zinc concentrations were significantly higher in the PZ and MNP groups as compared with the TZ group at endline (*P* = 0.003; Table 3). In the minimally adjusted models, the mean endline nail zinc concentrations were marginally (*P* = 0.055) higher among children in the PZ and MNP groups than in the TZ group, and in the fully adjusted models, these differences were statistically significant (*P* = 0.015) (Table 3). In the TZ group, exploratory statistical analyses indicated that the number of therapeutic zinc tablets consumed over the course of the intervention was marginally positively correlated with nail zinc concentration (*r*_s_ = 0.19, *P* = 0.081). Mean nail zinc concentration among children who consumed 0–10 therapeutic zinc tablets was 108.4 + 18.4 μg/g, compared with 114.3 + 13.4 μg/g among children who consumed > 11 therapeutic tablets over the course of the intervention period. In addition, the elapsed time since last therapeutic tablet consumption was negatively correlated with nail zinc concentration (*r*_s_ = − 0.26, *P* = 0.037).

**Table 3:** Effects of 32–40 weeks of supplementation with daily preventive zinc or daily multiple micronutrient powder on endline nail and plasma zinc concentrations among young Lao children^a^

Outcome	PZ		MNP		TZ		Minimally adjusted *p* value^b^	Adjusted *p* value
	n	Value^c^	n	Value	n	Value		
Plasma zinc concentration, μg/dL^d^	93	63.7 (60.9, 66.6)	77	60.1 (58.0, 63.9)	82	56.9 (54.3, 59.7)	0.003	
Nail zinc concentration, μg/g	95	115.8 (111.6, 119.9)	78	117.8 (113.3, 122.3)	83	110.4 (106.0, 114.8)	0.055	0.015

^a^*MNP* multiple micronutrient powder, *PZ* preventive zinc, *TZ* therapeutic zinc

^b^Minimally adjusted and adjusted models control for age and district of enrollment. Adjusted models include child underweight, breastfed in the previous month, minimum meal frequency, maternal education, and drinking water source

^c^Values represent mean (95% CI)

^d^Plasma zinc concentration data control for baseline values of the respective outcome and are adjusted for elevated acute phase proteins (CRP and AGP) based on Barffour et al. [12]. Plasma zinc concentration data are only presented for children for whom nail zinc concentration data are also available

Effect modification was explored for both hair and nail concentration outcomes with 12 modifiers. Statistically significant modification was identified for 3 of the 23 tests but further exploration showed no consistent patterns across treatment groups (data not shown).

### Associations Between Plasma Zinc Concentration and Hair and Nail Zinc Concentrations

Hair zinc concentrations were not associated with the concentrations of plasma zinc measured from the same time point, at either baseline (*P* = 0.62, *n* = 371) or post-intervention (*P* = 0.86, *n* = 406); adjusting for significant covariates did not alter the results. Similarly, endline nail zinc concentrations were not associated with endline concentrations of plasma zinc (*P* = 0.22, *n* = 257).

## Discussion and Conclusion

### Overview of the Main Results

In the present study, plasma zinc concentrations responded significantly to daily preventive zinc supplementation, regardless of whether the zinc was provided in the form of a single-nutrient supplement or MNP. On the other hand, we found that hair zinc concentrations did not respond to either form of preventive zinc supplementation. Nail zinc concentrations appeared to increase in response to daily preventive zinc supplementation, but these results are considered exploratory as the therapeutic zinc group rather than the placebo control group was analyzed. Finally, the prevalence of both low plasma zinc and low hair zinc concentrations are suggestive of a high risk of zinc deficiency in this population. However, concurrent measures of plasma and hair or nail zinc concentrations were not correlated with each other in the present analyses.

### Effects of the Intervention

The increase in plasma zinc concentration observed within the PZ and MNP groups in the present study are in agreement with the results of previous studies that have consistently shown a positive response of plasma zinc concentrations to zinc supplementation and similar doses of zinc in MNP as delivered in the current trial [3, 7, 27, 28]. The effects of previous studies of zinc supplementation on hair zinc concentration are not as straightforward. Although a systematic review and meta-analysis of three randomized controlled trials conducted in adults showed that hair zinc concentrations increased following zinc supplementation [7], results of the few randomized controlled trials conducted among infants and young children are inconsistent. One study conducted in Ethiopia found a significant increase in both plasma and hair zinc concentrations among stunted infants who received 10-mg Zn/day 6 times/week for 6 months [29]. In addition, both plasma and hair zinc concentrations increased significantly among pre-school aged girls, but not boys, who received zinc-fortified breakfast cereals in the USA [30]. However, two additional studies reported a significant increase in plasma zinc concentrations following zinc supplementation, but no impact on hair zinc concentrations [31, 32], similar to what was observed in the present study. Finally, four additional studies found no significant increase in hair zinc concentrations following zinc supplementation or fortification; although in these studies, plasma zinc concentrations were either not measured [33] or also did not respond to supplementation [34–36], making it difficult to interpret the hair zinc results.

We were able to identify only one study that examined the change in nail zinc concentrations in response to zinc supplementation. Among post-partum women in Egypt, nail zinc concentrations were significantly greater among those who received 10-mg Zn/day for 2 months compared with those who did not [37]. These results are consistent with the findings of the present study, which showed that nail zinc concentrations were greater among children who received daily preventive zinc supplements (7–10 mg of Zn/day) compared with those who received therapeutic zinc supplements for diarrhea. Although a direct comparison to the placebo control group would have been ideal, several exploratory analyses lend strength to these findings. Median (IQR) supplemental zinc consumption in the TZ group was < 10% of that consumed in the preventive zinc and MNP groups (TZ, 140 (20, 260) mg; PZ, 1512 (1376, 1610) mg; MNP, 2175 (1900, 2340) mg over the study intervention period), and nail zinc concentration was marginally associated with the number of therapeutic zinc tablets (i.e., total supplemental zinc consumption) in the therapeutic zinc supplementation group. Additional studies which measure the response of nail zinc concentrations to supplemental zinc interventions will be necessary to confirm these findings.

### Relationships among Zinc Biomarkers

In the present study, plasma and hair zinc concentrations were not correlated, which is consistent with the results of most [35, 38–40], but not all [37, 41, 42], of the studies that have examined the relation between these biomarkers. Plasma and nail zinc concentrations also were not correlated in this study. Plasma, hair, and nail zinc concentrations may reflect zinc exposure or status over different time frames with different sensitivities, which may lead to the observed lack of correlations between biomarkers. Plasma zinc concentrations reflect recent zinc intake [5]; they have been shown to respond rapidly (within 2–5 days) to short-term changes in zinc intake via supplementation and persist for the duration of the supplementation period [43]. Conversely, hair and nail zinc concentrations reflect the amount of zinc in the blood at the time of integument synthesis. For example, if the proximal 1–2 cm of hair is collected for analysis, hair zinc concentrations will reflect zinc exposure or status during the 4–8 weeks prior to sample collection [5]. Correlations with zinc content may have been obscured by the day-to-day and seasonal fluctuations in dietary zinc exposure and consequent changes in plasma zinc concentration.

### Determination of Cutoff Values for Low Hair and Nail Zinc Concentrations

There are currently no established reference values for hair zinc and nail zinc concentrations, which limits the use of these biomarkers as indicators of zinc status. A cutoff of 70 μg/g hair (< 1.07 μmol/g) has been commonly used to define zinc deficiency, based on associations with impaired growth, poor appetite, and hypogeusia among children 4–17 years in the USA [44]. However, among children 3 months–4 years in the aforementioned study, 36.6% had hair zinc concentrations below this cutoff, and correlations with anorexia and poor growth and hair zinc concentrations were only observed when hair zinc concentrations were < 30 μg/g hair [44]. In contrast, a study in Panama found that younger children (0–5 years) had higher hair zinc concentrations compared with older children (6–10 years) [45]. Since hair or nail and plasma zinc concentrations among infants and young children were not correlated in the present study, no additional information on an appropriate cutoff of hair and nail zinc concentrations to define zinc deficiency is available. Therefore, changes in nail zinc concentrations in response to an intervention reflect zinc exposure, but not necessarily zinc status.

### Strengths and Weaknesses

The randomized, placebo-controlled study design, standardization of biological sample collection and laboratory analysis, and adequate sample size lend strength to these findings. Plasma, hair, and nail samples were collected and analyzed based on procedures recommended by IZiNCG [9, 10], and appropriate certified reference materials (currently available for plasma and hair) were used to assess the accuracy and precision of the analytical methods. In addition, the study was implemented in a population with a high prevalence of zinc deficiency and stunting [12], and plasma zinc concentrations increased in response to supplementation among children who received preventive dispersible zinc tablets (7 mg/d) or MNP (10 mg Zn/d) daily for 9 months [12]. A primary limitation of this study is that no overall effects of functional outcomes of zinc status (i.e., linear or ponderal growth or diarrheal morbidity) were observed [12]. In addition, the observed increases in plasma and nail zinc concentrations were relatively small. Thus, the interventions’ effects on zinc status may have been limited, with hair zinc concentrations not sensitive enough to detect these changes. Additional limitations of this study include nail samples not being analyzed in the placebo control group, a lack of nail sample collection at baseline, and the combination of finger and toenail samples, which have different rates of growth, for the analytical samples.

## Conclusions

In the present study, zinc concentrations in plasma and nail, but not hair, responded to supplemental zinc intake. Thus, this study does not support the use of hair zinc as a biomarker of zinc exposure in young children. However, it provides preliminary evidence that nail zinc concentrations may respond to supplemental zinc interventions and supports the desirability of collecting additional data on this emerging biomarker.

## Electronic supplementary material

ESM 1 (PDF 111 kb)

## Abbreviations

AGP: α-1-acid glycoprotein
BOND: Biomarkers of Nutrition for Development
BRINDA: Biomarkers Reflecting Inflammation and Nutritional Determinants of Anemia
CRP: C-reactive protein
HFIAS: Household Food Insecurity Access Scale
IZiNCG: International Zinc Nutrition Consultative Group
LAZ: l Length-for-age z-score
MNP: Micronutrient powder
PDR: People’s Democratic Republic
PZ: Preventive zinc
TZ: Therapeutic zinc
WAZ: Weight-for-age z-score
WLZ: Weight-for-length z-score
