# Supplementary material for: Plasma and Nail Zinc Concentrations, But Not Hair Zinc, Respond Positively to Two Different Forms of Preventive Zinc Supplementation in Young Laotian Children: a Randomized Controlled Trial
Source: Biol Trace Elem Res. 2020 Apr 30;199(2):442–52. doi: 10.1007/s12011-020-02163-2 (PMC7746564; doi:10.1007/s12011-020-02163-2)
Supplement: Supplementary file 1 — (PDF 111 kb) [file 12011_2020_2163_MOESM1_ESM.pdf]

Supplemental Table 1. Micronutrients provided in multiple micronutrient powder (MNP)[a]

Nutrient	Chemical Form	Unit	MNP (1 g)
Vitamin A	Retinol acetate	µg RE	400
Thiamin (B1)	Thiamin mononitrate	mg	0.5
Riboflavin (B2)	Riboflavin	mg	0.5
Niacin (B3)	Niacinamide	mg	6
Vitamin B6	Pyridoxine hydrochloride	mg	0.5
Folic acid (B9)	USP	µg DFE	150
Vitamin B12	Cyanocobalamin, USP	µg	0.9
Vitamin C	Ascorbic acid	mg	30
Vitamin D	Cholecalciferol (D3)	µg	5
Vitamin E	dl-α-tocopheryl acetate	mg TE	5
Copper	Copper sulfate, anhydrous	mg	0.56
Iodine	Potassium iodate	µg	90
Iron	Ferrous fumarate	mg	6
Selenium	Selenium selenite	µg	17
Zinc	Zinc gluconate	mg	10

[a]DFE, dietary folate equivalent; MNP, micronutrient powder; RE, retinol equivalent; TE, tocopheryl equivalents
